# Global and local disturbances interact to modify seagrass palatability

**DOI:** 10.1371/journal.pone.0183256

**Published:** 2017-08-16

**Authors:** Rocío Jiménez-Ramos, Luis G. Egea, María J. Ortega, Ignacio Hernández, Juan J. Vergara, Fernando G. Brun

**Affiliations:** 1 Departament of Biology (Division of Ecology), Faculty of Marine and Environmental Sciences, University of Cádiz, Puerto Real, Spain; 2 Departament of Organic Chemistry, Faculty of Marine and Environmental Sciences, University of Cádiz, Puerto Real, Spain; Stazione Zoologica Anton Dohrn, ITALY

## Abstract

Global change, such as warming and ocean acidification, and local anthropogenic disturbances, such as eutrophication, can have profound impacts on marine organisms. However, we are far from being able to predict the outcome of multiple interacting disturbances on seagrass communities. Herbivores are key in determining plant community structure and the transfer of energy up the food web. Global and local disturbances may alter the ecological role of herbivory by modifying leaf palatability (i.e. leaf traits) and consequently, the feeding patterns of herbivores. This study evaluates the main and interactive effects of factors related to global change (i.e. elevated temperature, lower pH levels and associated ocean acidification) and local disturbance (i.e. eutrophication through ammonium enrichment) on a broad spectrum of leaf traits using the temperate seagrass *Cymodocea nodosa*, including structural, nutritional, biomechanical and chemical traits. The effect of these traits on the consumption rates of the generalist herbivore *Paracentrotus lividus* (purple sea urchin) is evaluated. The three disturbances of warming, low pH level and eutrophication, alone and in combination, increased the consumption rate of seagrass by modifying all leaf traits. Leaf nutritional quality, measured as nitrogen content, was positively correlated to consumption rate. In contrast, a negative correlation was found between feeding decisions by sea urchins and structural, biomechanical and chemical leaf traits. In addition, a notable accomplishment of this work is the identification of phenolic compounds not previously reported for *C*. *nodosa*. Our results suggest that global and local disturbances may trigger a major shift in the herbivory of seagrass communities, with important implications for the resilience of seagrass ecosystems.

## Introduction

Human activity is increasing, at the rate of 0.4% yr^−1^, the concentration of CO_2_ in the atmosphere, which is expected to double from preindustrial levels by the middle of this century [1]. Approximately 30% of CO_2_ emissions are absorbed by marine waters; therefore, marine species have to cope with increasing ocean acidification in combination with rising temperatures and other anthropogenic disturbance (i.e. a physical force, agent, or process, either abiotic or biotic, causing a perturbation in a ecological component or system; [2] and references therein), such as eutrophication. In this context, coastal vegetated ecosystems are one of the most threatened [3]. Within coastal vegetated habitats, seagrasses form the basis of one of the most species-rich and relevant ecosystems [4], but also one of the most threatened [5]. Seagrass communities are under threat from co-occurring global (e.g. rising temperatures, ocean acidification) and local disturbances (e.g. eutrophication), which are already acting together in many coastal areas with effects expected to increase in the near future [6,7]. How these changes will affect seagrass-herbivore interactions is one of the main questions for future seagrass research. We are far from being able to predict the outcome of interacting multiple perturbations (i.e. the response of an ecological component or system to disturbance by deviations in the values describing the properties of the component or system' relative to a specified reference condition; [2] and references therein) on the functioning of seagrass ecosystems. Until now, mesocosm and field experiments, mostly addressing only one factor at a time [8–10] but some with multifactorial designs [11–13], have shown the direct effects on plants in terms of growth and production, and changes in leaf content (N, P, non-structural carbohydrates) [8,9]. Even though several recent studies have analyzed the indirect effects of seagrass-herbivore interactions [14–17], few have addressed the outcome of interactions between more than two factors.

Changes in the growth or content of seagrass leaves due to global and local disturbances can exert a significant impact on seagrass communities because these changes may influence leaf palatability, and thus make plants more or less vulnerable to herbivores [18–20]. Eutrophication, one of the main local causes of seagrass decline, has been shown to affect the intensity of herbivory in seagrass ecosystems [21,22]. Under elevated nutrient concentrations, plants may increase the nutritional quality of their leaves (i.e. higher N content; [23–25]). However, the responses to this change may not always be straightforward. For example, Goecker et al. (2005) and Prado and Heck (2011) have shown that generalist herbivores (parrotfish and sea urchins) preferentially feed on leaves with higher nitrogen content, and lower leaf toughness and fiber content. However, others studies have reported the opposite trend [26–28].

At the global scale, increasing CO_2_ concentration may increase both seagrass primary productivity and leaf C/N ratio [10,29], which may reduce the palatability and nutritional quality of leaves. However, elevated CO_2_ concentrations in combination with ocean acidification has also been shown to down-regulate the production of C-based phenolic compounds in marine plants and thus enhance herbivore consumption of seagrass tissues [30], which may counterbalance the aforementioned positive effects on seagrasses. Warming temperatures are also predicted to increase rates of primary production [31], alter C/N ratios [32] and/ or the production of defensive secondary metabolites [33], which may further modify leaf palatability. Understanding how changes in the abiotic environment can modify the outcome of species interactions and being able to scale up these changes to the community level is of paramount importance for ecologists, particularly for interactions involving keystone species that have disproportionately large effects on community and habitat structure [34,35]. This challenge requires a multi-scale approach from the level of individual responses to that of populations, communities and the entire ecosystem [36], taking into account that the overall effect of multiple stressors can be non-additive [13,37].

*Cymodocea nodosa* (Ucria) Ascherson is a seagrass distributed across the Mediterranean Sea and adjacent eastern Atlantic coasts [38], including the Macaronesian archipelagos of Madeira and the Canaries, all the way down to Senegal in the western African coast [39,40]. These meadows constituted by *C*. *nodosa* are often the dominant vegetated communities on shallow soft substrates of the Andalusian coasts (south Spain), co-habiting with *Posidonia oceanica* or *Zostera noltei*. Interestingly, the experimental site of this work, Cádiz bay, is the unique place in Europe where the dominant vegetated community of *C*. *nodosa* (total extension of 1200 ha) is co-habiting with *Z*. *noltei* and *Zostera marina* populations [41]. These communities are highly productive and providing food and shelter for diverse invertebrates and fish assemblages [42,43]. Moreover, the trophic importance of *C*. *nodosa* as a food resource for herbivores were observed in several [44–46].

The main aims of this work are two-fold: to study the direct response of seagrasses to the main drivers of change in their communities, warming, acidification and eutrophication [5]; and to determine how these drivers indirectly affect the feeding preference of herbivores. We used a full-factorial experimental design to test for the effects of seawater temperature, pH and NH_4_^+^ concentration on traits of *C*. *nodosa* plants. After this, the plants were offered to *Paracentrotus lividus*, which is a type of purple sea urchin and a generalist herbivore of seagrass meadows, in individual feeding assays (i.e. just one food source) or in combined diet assays with a highly palatable species (*Ulva* sp.). Leaf traits and the consumption rate by sea urchins were measured to determine the main factors driving the feeding choice of this generalist herbivore.

## Materials and methods

### Sampling site and plant acclimation period

The seagrass *Cymodocea nodosa* Ucria (Ascherson) was randomly collected in the fall from large submerged meadows found in inner Cádiz Bay (36°28’09.08”N–06°15’04.64”W, South Spain; [41]). *C*. *nodosa* inhabits the shallow south-western area along the intertidal border and in continuous monospecific meadows in the subtidal zone, which are found at 0.4 m above and 0.5 m below the chart datum of lowest astronomical tide, respectively [47]. Plant biomass were selected in the field and collected from different patches in a large area (~150 × 150 m), to ensure the genetic independence of plants, since in this area the sexual reproduction is as high as clonal ones [48]. Plants were transported to the laboratory at the University of Cádiz, southern Spain, within 2 h of collection in an ice chest. Upon arrival, experimental plant units (EPUs) of *C*. *nodosa*, consisting of one vertical shoot with its first rhizome segment (i.e. first internode), were carefully selected for a healthy appearance. Then 20 EPUs of healthy *C*. *nodosa* were allocated to each of 24 transparent incubation chambers (1.5 L) containing natural sandy sediment. Chambers were connected to seawater reservoirs (65 L) (three chambers per reservoir) by silicon tubs. The seawater reservoir received sand-filtered seawater at a rate of 6 L d^−1^. Water in the incubation chambers was renewed by pumping water from their respective reservoir at a rate of 1.5 L d^-1^ in an open system. The water within chambers was homogenized by using air inlet drops.

After five days of plant acclimation to laboratory conditions (natural seawater, ambient light and temperature), seawater parameters (temperature, NH_4_^+^ and CO_2_ concentration) were modified in the different seawater reservoirs (65 L) in a full-factorial experimental design combining three of the main factors that are currently acting together in coastal areas (i.e. warming, acidification and NH_4_^+^ enrichment) in order to determine how these drivers affect the leaf traits of the temperate seagrass *C*. *nodosa*. A three-full factorial experiment was conducted during a month. Two temperature levels were used: (a) the local temperature treatment with ambient temperature ca. 22°C and (b) the high temperature treatment with seawater heated to 4°C above ambient levels until ca. 26°C. We also used two pH levels: (a) the current pH treatment of ca. 8.12, equivalent to ca. 415 ppm CO_2_ and (b) the forecasted pH treatment ca. 7.69, equivalent to future conditions of ca. 720 ppm CO_2_. Finally, we used two NH_4_^+^ levels: (a) the ambient NH_4_^+^ treatment with no added NH_4_^+^ and (b) the enriched NH_4_^+^ treatment where NH_4_^+^ was added to keep the concentration ca. 31 μM NH_4_^+^. All treatment levels were found in all combinations thus rendering a fully orthogonal design (8 treatment combinations in total). Each treatment combination was replicated 3 times resulting in a total of 24 experimental chambers. The levels of temperature and pH were selected according to the scenario forecasted by the Intergovernmental Panel on Climate Change (IPCC).

Temperatures were maintained by recirculating water through a heater (Tetra HT 100W). The pH in reservoirs with forecasted pH values were obtained by adding small amounts of HCl (0.01N) to the seawater until reaching the pH values linked to forecast ppm of CO_2_ (ca. 720 ppm total scale) (e.g. [49]). The NH_4_^+^ enrichment was obtained by daily addition of NH_4_^+^ to the reservoir from a NH_4_^+^ stock solution to keep the concentrations as close to the target concentration as possible (ca. 31 μM NH_4_^+^). The ammonium addition corresponded to ca. 700 μmol g FW^−1^ d^−1^ in enriched NH_4_^+^ chambers. The concentration of ammonium was monitored according to Invers *et al*. (2004) [50] every two—three days in all chambers and daily in all reservoirs. Water samples were collected ten minutes after addition of ammonium. Seawater temperature and pH in the incubation chambers were measured daily at sunrise and sunset. The average salinity in the chambers during the experimental period was 30.08 ± 0.11. Light period was 16:8 h (light:darkness) with an instant photon flux of 325 μmol photon m-^2^ s^-1^. S1 Table lists the total alkalinity, pH, temperature, as well as the dissolved inorganic carbon in the seawater in the incubation chambers.

At the end of the cultivation period (1 month), plant samples from each treatment were split: 5 EPUs from each chamber were selected for structural analyses (i.e. length, width and thickness) while another 5 EPUs from each chamber were collected for biomechanical measurements (i.e. leaf fracture properties) and once measured, immediately frozen at –18°C for complementary nutrient analyses (i.e. N, C and fiber content). Additionally 6 g of fresh leaf tissue from each chamber were immediately frozen at -80°C for chemical analyses (i.e. natural product determination). The remaining plants were kept alive in a 24 L tank with aeration and natural sandy sediment for feeding trials.

### Structural and nutritional analyses

Leaf structural measurements were taken from healthy and fully-developed leaves from 5 EPUs per each chamber at the end of cultivation period. Each sample was freeze-dried and pulverized in a ball-grinder. Leaf thickness was measured 2 cm above the ligule using a thickness gauge (mm). The total C and N content in tissue samples was measured using a Perkin-Elmer 2400 elemental analyser. Nutrient content of tissues were examined in molar ratios, and the final results were based on dry biomass. Intracellular concentrations of NH_4_^+^ were measured on duplicated leaf samples from each aquarium. Samples were rinsed in deionized water and ca. 0.5 g (FW) of the tissue was ground in 20 ml of boiling deionized water [51]. Samples were sonicated for 10 min and then centrifuged for 20 min at 5,000 g. Finally, the concentration of NH_4_^+^ was measured in the supernatant following Grasshoff et al. (2007)[52].

The dry biomass of each sample was used to determinate fibre content using a method modified from Van Soest et al. (1991) [53]. Namely, approximately 30 mg samples of dry biomass were boiled in 2 ml of neutral detergent for 1h. Subsequently, these samples were centrifuged (5 min at 2,500 g) and the resulting pellets were washed with distilled water (x2), ethanol (x2) and acetone (x1), with a centrifugation step as indicated above following each washing step. The final pellet, which was free of non-cell wall components and chlorophyll, was dried overnight in an oven (60°C) and subsequently weighed. Fiber content of seagrass tissues was obtained from the difference in mass before and after the procedure, and expressed as a percentage of dry biomass.

### Natural product analyses

#### Extraction procedures

Samples (0.5–1.3 g DW) were extracted with MeOH (5mL x 3) under maceration with shaking and sonication for 3min. The resulting slurry was centrifuged for 5 min and the supernatant taken to dryness under vacuum evaporation. The obtained residue (c.a. 50 mg) was partitioned between H_2_O and Et_2_O, and then n-BuOH (3 x 2 mL). The butanolic extracts were used to analyse the natural products.

#### UPLC-MS analysis of compounds

Identification of compounds was carried out using an ACQUITY Ultra Performance LC system equipped with a photodioide array detector with a binary solvent system (Waters corporation, Milford, MA, USA) with a mass detector Xevo G2 Q-TOF mass spectrometer (Waters, Manchester UK) equipped with an electrospray ionization (ESI) source operating in negative mode (Mass Spectrometry Facilities, SC-ICYT, University of Cádiz). Separation of compounds was carried out using a UPLC BEH C18 column (1.7 μm, 2.1 x 50 mm, Waters) at 25°C. For UPLC-MS analysis, each butanolic extract was suspended in H_2_O and a solution of quercetin (Sigma-Aldrich) was added until final concentrations of 1500–2000 μg mL^-1^ for the extract and 2.5 μg mL^-1^ for quercetin were reached (internal standard). The volume of injection was set at 5 μL and the flow rate 0.4 mL min^-1^. The solvents for the mobile phase were H_2_O + 0.1% of formic acid (A) and CH_3_CN (B). The following gradient was used: 0.00 min 90% A, 0.00–3.50 min 55.0% A (linear), 3.50–4.20 min 90.0% A (linear), and 4.20–5.00 min 90.0% A (isocratic). The analyses were carried out using a full-scan, data-dependent MS scanning from m/z 100–1000. The mass spectrometer was operated in negative ion mode. The following optimized MS conditions were used: source temperature 120°C, capillary voltage 3000 V, cone voltage 20V, desolvation temperature 350°C, and desolvation gas (nitrogen) flow rate 850 L h^-1^. Characterization of the single components was achieved using the retention times and the accurate molecular masses. Leucine-enkephalin was used as the reference compound. The [M-H]^-^ ions were detected at 554.2615 Da during an analysis performed within ESI—MS accurate mass experiments, which were permanently introduced via the LockSpray channel using a Hamilton pump. The lock mass correction was ±1.000 for the mass window. Collision-induced fragmentation experiments were performed using argon as the collision gas, with voltage ramping cycles from 0.3 to 2 V. Characterization of the single components was carried out via the retention time and the accurate molecular masses. Each compound was optimized for its estimated molecular mass in the negative mode, before and after fragmentation. The data obtained from UPLC—MS were subsequently entered into MassLynx 4.0ChromaLynx Application Manager software (Waters). For UPLC-MS/MS analysis, the following parameters were used: source temperature 150°C, capillary voltage 3000 V, cone voltage 20 V, trap collision energy 20–40 eV, and mass scan range m/z 50–1200 Da. The elution of compounds from the column was simultaneously monitored by a photodiode array (PDA) detector at λ 300–450 nm.

The quantification of the phenolic compounds (see S1–S3 Figs and S2 Table) was performed using external calibration curves of a reference compound selected based on the principle of structure-related target analyte/standard (chemical structure and functional group). The calibration curve for rutin (1) was used to quantify compounds 4 and 6, in addition to being used for its own quantification. The calibration curve for quercetin-3-ß-D-glucoside (2) was used to quantify compounds 3, 5 and **7**, in addition to being used for its own quantification. As an internal standard, quercetin was used. All determinations were done in triplicate (*n* = 3).

### Biomechanical analyses

The biomechanical properties of *C*. *nodosa* leaves were measured with an Instron testing machine (model 5542) and BlueHill^®^ software (v.2.18). We sampled the first outermost fully-developed leaf from the selected EPUs (five leaves per chamber, normally the second youngest leaf), and a portion of the leaf blade was cut 4 to 5 cm above the ligule for testing. The leaves were measured within 1d of sampling and the specimens were tested in the same sequence as they had been collected so that the time of storage was similar among samples and treatments. Leaf-fracture properties were evaluated by two tests (cutting and tearing tests) and expressed at 2 levels: (1) total quantity of force needed to cut or tear a single leaf blade, which depends on the leaf size and its mechanical properties at the material level (F_TA_, N); (2) material biomechanical traits, normally called ‘material properties’, which are inherent properties of the material (F_TS_; N mm^-2^). Regarding the ecological significance of these traits, whole-leaf biomechanical traits indicate the force needed in absolute terms to cut or tear a single leaf blade by herbivores, whereas material properties show the invested work or force required to ingest an amount of material, giving an idea of the cost-efficiency of the feeding process [54]. The cutting test measures the force required for foliar breakage [55,56]. During the test, a force to displacement curve was monitored. Since the whole leaf was cut transversally during the test, the force exerted to cut the lamina also included the leaf veins [54]. The tensile (tearing) tests were conducted along the long axis of the leaf. The F_TA_ (N) and the F_TS_ (N mm^−2^) were obtained from the force-displacement curve and the size traits.

### Collection of consumers

Sea urchins (*Paracentrotus lividus*) were collected from La Caleta, Cádiz (SW Spain, 36°31’39”N; 6°18’46”W). The most stable populations of *P*. *lividus* inhabit this place and therefore this area was chosen for the extraction of individuals. After authorization from the local environmental division, 150 individuals of sea urchins were collected from a depth of 2 meters. Harvesting was carefully carried out by snorkelling to avoid damaging the animals. Sizes ranged from 3–5 cm in diameter. Collected individuals were kept in coolers with seawater and transported to the laboratory. Upon arrival, sea urchins were placed in aerated tanks (4 tanks of 30 liters each, 37 individuals in each tank) and were fed with the macroalgae *Ulva* sp. for 3 days until the beginning of the experiment to allow acclimation to laboratory conditions.

### Experimental set-up for feeding assays

To experimentally examine how the drivers of change (i.e. elevated temperature, pH level and nutrient input) affect the feeding preference of the sea urchin *Paracentrotus lividus*, we conducted a suite of no-food choice and food choice feeding assays between *Cymodocea nodosa* from each of the experimental chamber and *Ulva* sp. *Ulva* sp. was used to check the health status of the sea urchins during the different assays (it is voraciously consumed by healthy sea urchins), and as an indicator of the food preference by sea urchins [57]. Feeding assays were run in a temperature controlled climate room set at 20°C, where sea urchins were placed in aquaria (individual volume ≈ 20 L) in a closed flow-through system. The aquaria were illuminated by lamps with cool fluorescent tubes (T5 High Output Blau Aquaristic aquarium color extreme fluorescents) in a 8:16h (light:darkness) because *P*. *lividus* usually exhibits nocturnal activity [57]. Aeration pits were placed in all aquaria to ensure adequate mixing of water and air. Sea urchins were acclimated for 72 hours prior to starting each assay, during which time they were fed the palatable alga *Ulva* spp. to avoid any interference of previous food or starving on their foraging behaviour.

Two types of feeding assays were conducted: i) individual diet (ID, no food choice assays) and ii) combined diet (CD, food choice assays). In the individual diet assays, we offered 6 g of *C*. *nodosa* from each of the experimental chambers (8 treatments per 3 replicates, *n* = 24). In the combined diet assays, we offered 3 g of *C*. *nodosa* from each of the experimental chambers (8 treatments per 3 replicates, *n* = 24) and 3 g of *Ulva sp*., maintaining the same sea urchin—food ratio as used in the individual diet assays (6 g in total). All feeding assays (*n* = 48) were run with 3 individual sea urchins, which had been starved for 24 h prior to the beginning of the feeding experiment. Each feeding assay was run for 24 h under constant temperature (20°C) and aeration. We also used control containers during the assays to account for potential changes not resulting from grazing (i.e., seagrass not exposed to herbivores). These did not change in terms of fresh biomass and were thus not considered in the statistical analyses.

After 24 h, the remaining biomass was collected from the tanks, dried and weighed. The results of herbivory were expressed as biomass consumption per individual (i.e. sea urchin) per day. Sea urchins were weighed after each assay to control for their size.

### Statistical analyses

Prior to any statistical analysis, data were checked for normality (Shapiro-Wilk normality test) and homoscedasticity (Bartlett test of homogeneity of variances). Treatment differences were analysed using a three-way ANOVA: treatment factors were temperature, pH and NH_4_^+^ enrichment; response factors were C/N ratio, internal NH_4_^+^, fiber content, biomechanical properties, natural product content and consumption rate. Consumption rates in the no-choice assays (individual diet) were analysed by means of a three-way ANOVA. Differences in the two-choice assays (combined diet) between *Ulva* sp. and *C*. *nodosa* were analysed using a *t*-test (S3 Table). On the other hand, to find differences on C. nodosa consumption in the different treatments, a three-way ANOVA (with three fixed factors: temperature, pH and NH_4_^+^ enrichment) were performed. When significant differences were found, a Tukey post-hoc test was applied.

When statistical differences were not found using the three—way ANOVA, but differences between treatments were large, it may indicate the existence of a power issue because of the limited sample size (*n* = 3), and then a statistical meta-analysis of the effect size was used to avoid the possibly misleading influence of sample size. While null hypothesis significance testing only informs about the probability of an observation, the presentation of the effect size along with its standard error (SE) provides the two most important pieces of statistical information for biologists: the magnitude estimate of an effect of interest and the precision of that estimate [58]. Thus, if there are non-significant differences but large effects, it may suggest further research with greater power [59]. To estimate the effect size of the parameters under study, the Hedges' d metric was chosen [60], as it is an unbiased estimator that provides a better estimate for small sample sizes. The effect size was presented as Hedges’ d ± asymptotic standard error for the effect size according to [58]. Hedges' d metric values above 0 indicate a positive effect, bellow 0 indicate a negative effect, and equal to 0 indicates no effect on the parameter under investigation. The bigger the number either on the positive or negative direction tells about the magnitude of the effect. Cohen, 1988 [61] has proposed ‘conventional’ values as benchmarks for what are considered to be ‘small’, ‘medium’, and ‘large’ magnitude of the effects (d = 0.2, 0.5, 0.8, respectively) [58].

To test for interactions among factors, we used pairwise comparisons when significant interaction terms were detected [62]. Data are presented as mean ± SE. The significance level (α) was set in all tests at 0.05. Pearson’s correlation analysis was used to test the possible correlations between the consumption rate of *C*. *nodosa* in the individual and combined diet assays and the measured properties of *C*. *nodosa* leaves. Statistical analyses were computed with R 3.0.2 (R Development Core Team 2013).

## Results

### Leaf structural and nutritional traits

**Fig 1:**
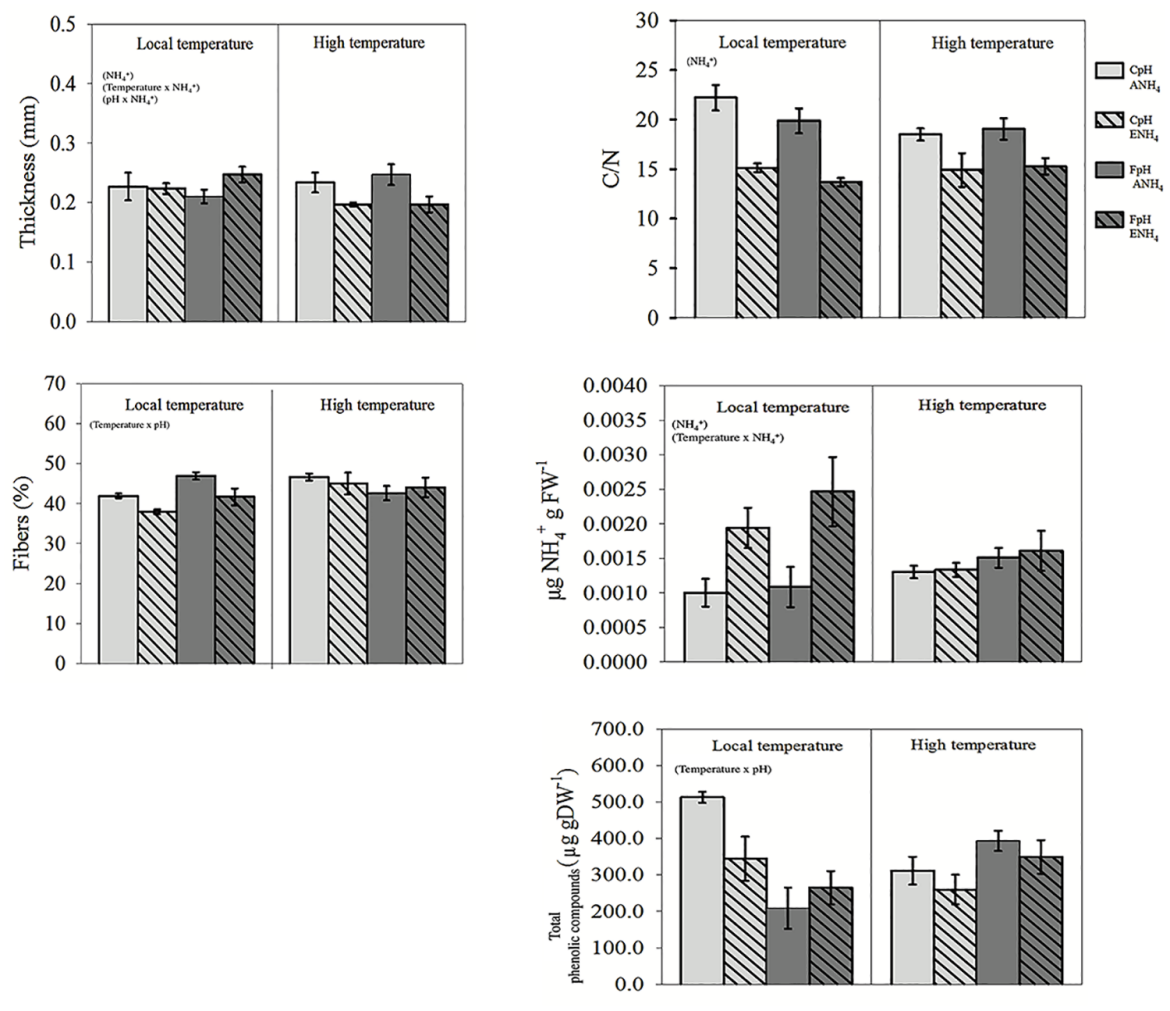
Leaf structural and nutritional traits of *C*. *nodosa* exposed to different temperature (local 22°C vs high 26°C), pH levels (current, CpH, vs forecasted, FpH) and NH_4_^+^ concentration (ambient ammonium, ANH_4_^+^, vs enrichment, ENH_4_^+^). Data are expressed as mean ± SE of thickness, fiber content, C/N ratio, concentration of ammonium and concentration of phenolic compounds. Factors resulting in significant differences in the three-way ANOVA are shown in parentheses (*p*<0.05).

**Fig 2:**
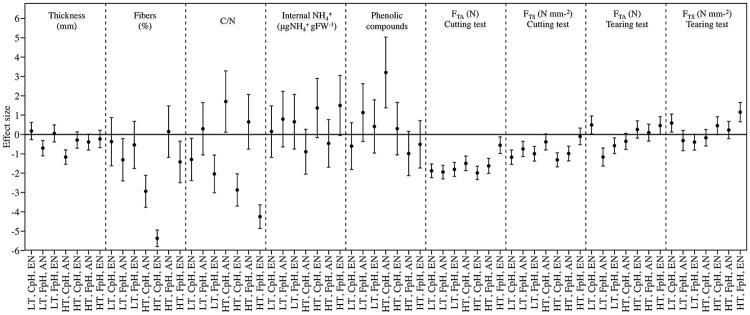
Effect size (*n* = 5) of *Cymodocea nodosa* leaf traits exposed to different temperature (local 22°C vs high 26°C), pH levels (current, CpH, vs forecasted, FpH) and NH_4_^+^ concentration (ambient ammonium, ANH_4_^+^, vs enrichment, ENH_4_^+^). Error bars indicate the 95% confidence intervals of thickness (mm), fiber content (%), C/N ratio, concentration of internal ammonium (μgNH_4_^+^·gFW^-1^), concentration of phenolic compounds (μg·gDW^-1^, *n* = 3), whole-leaf biomechanical traits (F_TA_, N) and absolute force-to-tear (F_TS_, N·mm^-2^) for cutting and tearing test.

Leaf traits varied significantly among the treatments (Fig 1). Regarding the structural properties of *Cymodocea nodosa* leaves, both elevated temperature and lower pH levels significantly increased fibre content, with an effect size of about -2.38 and -5.38 for treatments combining ambient and enrichment NH_4_^+^ concentration respectively, thereby potentially reducing palatability, while combined elevated temperature and NH_4_^+^ enrichment reduced fibre content (Fig 1), with a negative effect size of about -5.38 and -1.42 combining low and high pH level respectively (Fig 2). NH_4_^+^ enrichment also significantly increased leaf thickness (*p*<0.01). In addition, NH_4_^+^ enrichment positively affected the nutritional quality of *C*. *nodosa* leaves by decreasing the C/N ratio and increasing internal NH_4_^+^. In contrast, elevated temperature resulted in the highest C/N ratio (22.2 ± 1.28) as well as the lowest internal NH_4_^+^ concentration (1.0 ± 2x10^-5^ μg NH_4_^+^ gFW^-1^), both of which could negatively affect palatability. However, plants subjected to the combination of all three factors (higher temperature, lower pH and higher ammonium) had the highest internal NH_4_^+^ concentration with a positive effect size value of 1.50.

### Natural products

The analyzed samples showed the presence of the following phenolic compounds (see S2): rutin (1), quercetin-3-ß-D-glucoside (2), quercetin-3-ß-D-glucoside-6´´-acetate (3) isorhamnetin-3-ß-rutinoside (4), isorhamnetin-3-ß-D-glucoside (5), and two isprenylflavonols (6) and (7). Although compounds 2 and 5 have been previously reported in *C*. *nodosa* [63], this is the first time that compounds 1, 3, 4, 6 and 7 have been found in this plant, to the best of our knowledge. Leaves from plants acclimated to high temperature contained the highest concentration of phenolic compounds when compared with the rest of treatments (*p* < 0.05), with a positive effect size of 3.20 combining low pH level and ambient NH_4_^+^ concentration. However, when high temperature was combined with lower pH levels, the lowest concentration of phenolic compounds was recorded (Fig 1). Nutrient enrichment, however, did not result in any differences in phenolic compounds among the treatments in both three-way ANOVA test (Fig 1) and effect size analyses (Fig 2).

### Biomechanical traits

**Fig 3:**
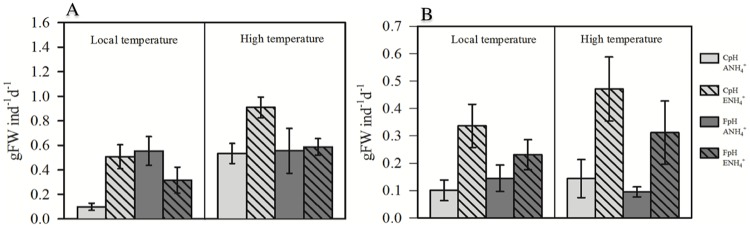
Consumption rates of *Cymodocea nodosa* by sea urchins in combined [A] and individual diet treatments [B] (g fresh weight [FW] ind^-1^ d^-1^; mean ± SE. *C*. *nodosa* had been exposed to different temperature (local temperature vs high temperature), pH levels (current pH, CpH, vs forecasted pH, FpH) and NH_4_^+^ concentration (ambient ammonium, ANH_4_^+^, vs enrichment, ENH_4_^+^).

Leaves from control plants (local temperature, current pH and ambient NH_4_+) were the most resistant (i.e. higher absolute force, F_TA_) in cutting tests and also had the highest tensile strength (i.e. higher specific force, F_TS_) (Tables 1 and 2). Both, three-way ANOVA test (Table 2) and effect size analyses (Fig 3) showed two-way interaction effects between lower pH levels and the others factors (increased temperature and NH_4_^+^ supply), which caused a significant decrease in whole-leaf biomechanical properties (i.e. lower values of F_TA_, Table 1), and as a consequence, plants showed lower resistance under such factor combinations. However, the three-way interaction between all factors increased the resistance of leaves in comparison to the other interactions, with an effect size of 0.46 for F_TS_ in tearing test (Fig 2). Regarding the tearing test, plants subjected to the combination of the three factors together also showed the most resistant leaves, as well as those with the highest tensile strength.

**Table 1:** Biomechanical traits of *Cymodocea nodosa* leaves exposed to different temperature (local 22°C vs high 26°C), pH levels (current, CpH, vs forecasted, FpH) and NH_4_^+^ concentration (ambient ammonium, ANH_4_^+^, vs enrichment, ENH_4_^+^).

	Cutting test		Tearing test	
	F_TA_ (N)	F_TS_ (N mm^-2^)	F_TA_ (N)	F_TS_ (N mm^-2^)
Local Tª, CpH, ANH_4_^+^	0.564 ± 0.083	0.880 ± 0.12	1.487 ± 0.144	0.995 ± 0.101
Local Tª, CpH, ENH_4_^+^	0.297 ± 0.077	0.382 ± 0.106	1.693 ± 0.075	1.130 ± 0.113
Local Tª, FpH, ANH_4_^+^	0.286 ± 0.060	0.533 ± 0.122	1.025 ± 0.105	0.741 ± 0.151
Local Tª, FpH, ENH_4_^+^	0.309 ± 0.048	0.497 ± 0.077	1.306 ± 0.066	0.825 ± 0.067
High Tª, CpH, ANH_4_^+^	0.279 ± 0.034	0.417 ± 0.054	1.610± 0.112	1.080 ± 0.081
High Tª, CpH, ENH_4_^+^	0.360 ± 0.096	0.665 ± 0.152	1.358 ± 0.12	0.892 ± 0.075
High Tª, FpH, ANH_4_^+^	0.345 ± 0.052	0.501 ± 0.076	1.550 ± 0.124	1.014 ± 0.081
High Tª, FpH, ENH_4_^+^	0.516 ± 0.113	0.798 ± 0.167	1.813 ± 0.243	1.383± 0.136

Values are means ± SE of replicates. For cutting and tearing tests., Whole-leaf biomechanical traits = F_TA_ (N), absolute force-to-tear; Material biomechanical traits = F_TS_, specific force-to-tear (N mm^-2^).

**Table 2:** Results of the three-way ANOVA for biomechanical properties of *Cymodocea nodosa* leaves exposed to different temperature (local 22°C vs high 26°C), pH levels (current, CpH, vs forecasted, FpH) and NH_4_^+^ concentration (ambient ammonium, ANH_4_^+^, vs enrichment, ENH_4_^+^).

Cutting test	df	MS	F	*p*-value
**F**_**TA**_ **(N)**				
Temperature	1	0.224	3.558	0.71
pH	1	0.106	1.687	0.84
NH_4_^+^	1	0.411	6.529	0.42
Temperature: pH	1	0.637	10.127	**0.035**
Temperature: NH_4_^+^	1	0.011	0.178	0.11
pH: NH_4_^+^	1	0.0003	0.005	**0.02**
Temperature: pH: NH_4_^+^	1	0.03	0.486	0.38
**F**_**TS**_ **(N mm**^**-2**^**)**				
Temperature	1	0.291	3.934	0.59
pH	1	0.011	0.152	0.79
NH_4_^+^	1	0.487	6.587	0.095
Temperature: pH	1	0.939	12.706	0.29
Temperature: NH_4_^+^	1	0.093	1.269	0.061
pH: NH_4_^+^	1	0.017	0.231	**0.003**
Temperature: pH: NH_4_^+^	1	0.052	0.708	0.62
**Tearing test**				
**F**_**TA**_ **(N)**				
Temperature	1	0.04	0.141	0.064
pH	1	0.01	0.039	0.199
NH_4_^+^	1	0.20	0.644	**0.013**
Temperature: pH	1	1.46	4.676	**0.0024**
Temperature: NH_4_^+^	1	0.81	2.589	0.67
pH: NH_4_^+^	1	1.78	5.69	0.94
Temperature: pH: NH_4_^+^	1	0.244	0.781	0.48
**F**_**TS**_ **(N mm**^**-2**^**)**				
Temperature	1	0.1003	0.298	0.052
pH	1	0.0229	0.068	0.7
NH_4_^+^	1	0.968	2.876	**0.013**
Temperature: pH	1	0.381	1.132	**0.0008**
Temperature: NH_4_^+^	1	1.226	3.64	0.26
pH: NH_4_^+^	1	3.063	9.094	0.63
Temperature: pH: NH_4_^+^	1	0.085	0.253	0.4

For abbreviations used in the cutting and tearing tests, see caption for Table 1. Bold font indicates significant differences (*p*<0.05).

#### Consumption rate of *Cymodocea nodosa*

**Fig 4:**
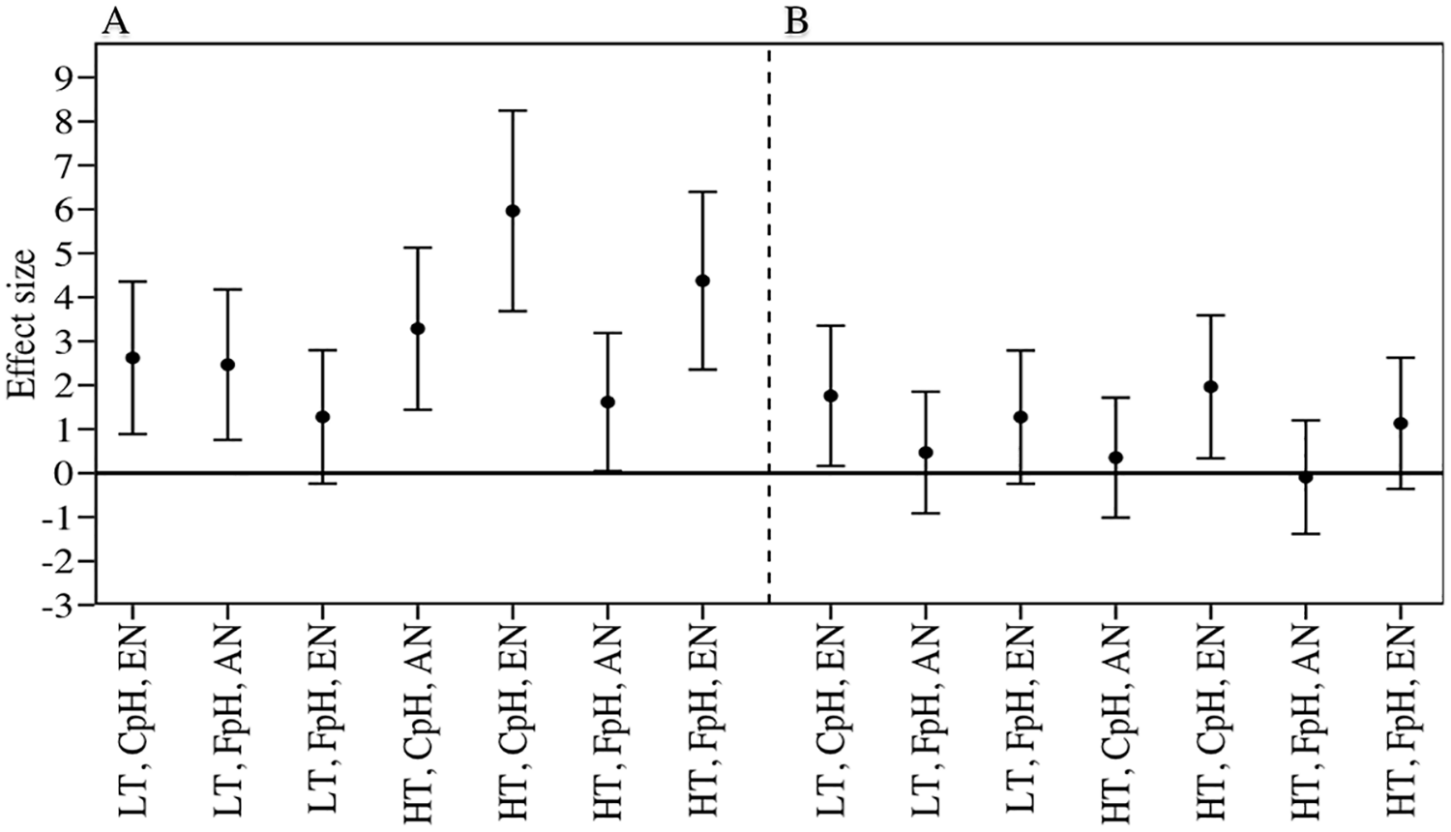
Effect size (*n* = 3) of *Cymodocea nodosa* consumption rate by sea urchins in combined [A] and individual diet treatments [B] (g fresh weight [FW] ind^-1^ d^-1^. Error bars indicate the 95% confidence intervals. *C*. *nodosa* had been exposed to different temperature (local temperature vs high temperature), pH levels (current pH, CpH, vs forecasted pH, FpH) and NH_4_^+^ concentration (ambient ammonium, ANH_4_^+^, vs enrichment, ENH_4_^+^).

The experimentally manipulated factors (higher temperature, lower pH levels and higher NH_4_^+^ concentration) increased the consumption rate of sea urchins with respect to the control treatments (Fig 3 and Table 3), both individually and in combination with other factors. In the combined diet treatments, only elevated temperature resulted in significantly increased consumption rates (p < 0.05). However, when sea urchins had only seagrass leaves to consume (individual diet), NH_4_^+^ supply was the variable determining feeding preference, with a positive effect size of 1.75 and 1.96 combining low pH level in high and local temperature treatments (Fig 4). Control plants were consumed at slower rates by sea urchins in both types of diet assays.

**Table 3:** Results of the three-way ANOVA on response of herbivore consumption rate to treatment of *Cymodocea nodosa* leaves exposed to different temperture (local 22°C vs high 26°C), pH levels (current, CpH, vs forecasted, FpH) and NH_4_^+^ concentration (ambient ammonium, ANH_4_^+^, vs enrichment, ENH_4_^+^).

**Combined diet**				
	Df	MS	F	(*p*-value)
Temperature	1	0.462	14.18	**0.002**
pH	1	0.0005	0.015	0.9
NH_4_^+^	1	0.125	3.846	0.68
Temperature: pH	1	0.119	3.654	0.07
Temperature: NH_4_^+^	1	0.021	0.649	0.43
pH: NH_4_^+^	**1**	0.368	11.297	**0.004**
Temperature: pH: NH_4_^+^	1	0.034	1.054	0.32
**Individual diet**				
	Df	MS	F	(*p*-value)
Temperature	1	0.016	0.955	0.34
pH	1	0.027	1.602	0.22
NH_4_^+^	1	0.28	16.529	**0.0009**
Temperature: pH	1	0.008	0.472	0.5
Temperature: NH_4_^+^	1	0.018	1.093	0.31
pH: NH_4_^+^	1	0.025	1.494	0.24
Temperature: pH: NH_4_^+^	1	0.0005	0.034	0.85

Bold numbers indicate significant differences (*p*<0.05).

In all the feeding assays, *Ulva* sp. was consumed at significant higher rates than the seagrass in both the individual (1.70 ± 0.37 gFW ind^-1^ d^-1^) and combined diet assays (1.09 ± 0.17 gFW ind^-1^ d^-1^ as a mean of all treatments, S3 Table).

### Correlation of consumption rates and leaf traits

In the pairwise comparisons between leaf traits (i.e. structural, nutritional, biomechanical and chemical) and consumption rates (for individual and combined diets), we found a significant positive correlation between leaf N content and consumption rate in the individual assays (*p*<0.05; Table 4). Also, a negatively correlation between fiber content and consumption rate was found in the combined diet assays (*p*<0.05). In both feeding assays (individual and combined diet), F_TS_ (in cutting tests) was negatively correlated to consumption rates (*p*<0.05).

**Table 4:** Pairwise Pearson's correlation coefficients between herbivore consumption rate and structural, nutritional, biomechanical and chemical traits of leaves.

		Structural traits			Nutritional traits		Cutting test		Tearing test		Chemical traits
		C	NDF	Th	N	NH_4_^+^ internal	F_TA_	F_TS_	F_TA_	F_TS_	Phenolic compounds
Combined diet	Correlation	-0.28	-0.49	-0.19	0.16	0.27	-0.24	-0.54	0.29	0.22	-0.26
	*p*-value	0.19	**0.015**	0.37	0.44	0.19	0.24	**0.006**	0.17	0.3	0.26
Individual diet	Correlation	-0.21	-0.29	-0.11	0.39	0.3	-0.2	-0.54	0.27	0.13	0.06
	*p*-value	0.33	0.16	0.59	**0.04**	0.15	0.34	**0.006**	0.207	0.55	0.79

Correlations between averaged values for each treatment (*n* = 3). Statistical significance indicated by bold font (*p*<0.05). Structural traits: C, leaf carbon content; NDF, leaf fibre content; Th, leaf thickness. Nutritional leaf traits: N, leaf nitrogen content; internal NH_4_^+^, internal ammonium. Whole-leaf biomechanical traits: F_TA_, absolute force-to-tear (N). Material leaf biomechanical traits: F_TS_, specific force-to-tear (N mm^-2^). Chemical traits: Phenolic compounds (μg gDW^-1^).

## Discussion

The assayed factors temperature (local and elevated temperature, 22°C *vs* 26°C), pH levels (current and forecasted pH, 8.12 vs 7.69) and NH_4_^+^ concentration (ambient and enriched NH_4_^+^ concentration, 0 and 31 μM), acting independently or in combination, modified the structural, nutritional, biomechanical and chemical properties of *Cymodocea nodosa* leaves, thereby affecting their palatability. Plant acclimated to NH_4_^+^ enrichment alone and in combination with the other two factors showed simultaneously higher N content, internal NH_4_^+^ content in leaves and reduced resistance of the leaves (lower F_TA_) (Fig 1). Moreover, lower pH levels decreased the resistance of leaves and concentration of phenolic compounds (Figs 1 and 2 and Table 2). As a consequence, under such conditions, leaf tissues may be more palatable and, therefore, more vulnerable to consumption since herbivores typically adjust their consumption rates relative to the nutritional content of their food source [64]. Thus, we found in both types of feeding assays that the consumption rates of *C*. *nodosa* leaves acclimated to predicted future levels of the three factors (independently or in combination) was higher than those recorded for control plants, in both three-ways ANOVA (Fig 3) and effect size analyses (Fig 4). Therefore, it is expected that both global (i.e. increase in temperature and acidification) and local shifts (i.e. eutrophication) will trigger an increase in the grazing of seagrass tissues.

Several studies have demonstrated a direct relationship between leaf nitrogen content and grazing [19,65]. However, others authors have found opposite patterns [26–28]. This suggests that the decision by herbivores to feed on one species or another does not depend only on one factor but may depend on a combination of factors. Here, we present the first study that analyzes a broad spectrum of leaf properties that define plant palatability under the main drivers of change in seagrass ecosystems (warming, acidification and eutrophication) [5]. First, we showed that NH_4_^+^ enrichment increased nutritional quality (i.e. higher N content and lower C/N ratios) and enhanced seagrass susceptibility to consumption by sea urchins, showing a positive correlation with N content in leaves in accordance with general predictions [64,66]. We also recorded negative correlations between consumption rate and responses expected to decrease grazing [67], such as structural (i.e. thickness, fiber and carbon content), chemical (i.e. phenolic compounds) and biomechanical defence traits (resistance of leaves) (Table 4). Some studies analysing plant traits that mediate feeding choices of seagrasses by consumers have observed structural traits [14,20,68] and chemical compounds acting to deter feeding on seagrass leaves [69]. Others have hypothesized that biomechanical traits [41] may affect the quality of a plant as suitable food, with increased tissue resistance making leaves less palatable because of the greater effort needed to tear off and chew tissue. Here, we present experimental findings on changes in biomechanical properties under a global change scenario, their relations to other leaf properties and their influence on feeding decisions by a generalist herbivore. We observed a reduction in leaf resistance (lower F_TS_ and F_TA_ in cutting test analyses) under the acclimation of different global factors such as the combination of high temperature and low pH levels or local factors such as NH_4_^+^ enrichment. Therefore, these results in combination with previous findings demonstrate how nutrient enrichment is the main driver of seagrass consumption by changing not only nutritional quality [64] but also biomechanical traits, enhancing seagrass susceptibility to consumption.

However, NH_4_^+^ enrichment was not the only trigger for changes in foliar properties and, thus, the consumption of plants. High temperature in combination with the other two factors (i.e. low pH and nutrient enrichment) acted to reduce the resistance of leaves as well as lowering C/N ratios, thickness and phenolic compounds, making plants more palatable to herbivores. Accordingly, studies have found that warming temperatures increased plant susceptibility to herbivores (e.g., C/N ratio [32] but also increased secondary defense metabolites [33]). Others have shown a reduction of secondary metabolites under the scenario of ocean acidification, in *C*. *nodosa* leaves and *Posidonia oceanica* seedlings [30,70], suggesting enhanced susceptibility of seagrass to grazing pressure. A notable point of this work is the identification of phenolic compounds not previously reported for *C*. *nodosa* (see S2 Fig). Among the several metabolites that plants produce, we focused attention in the phenolic compounds in particular because they bear a variety of deterrent and ecological functions [71]. These compounds, characterised by a carbon-based structure, with one or more hydroxyl groups bounded to an aromatic ring, have been the main products found in *C*. *nodosa* (e.g. caffeic acids and flavonoid glucosides such as quercetine and isorhamnetine monoglucosides; [63,72]). Moreover, it is known that phenolic acids and condensed tannins are commonly used by marine macroalgae to reduce palatability or increase toxicity to herbivores [71,73–77]. Interestingly, past studies have observed that low pH conditions could alter the concentration of these compounds in *C*. *nodosa*, reporting a loss of simple and polymeric phenolics in their leaves [30]. While we also detected a decrease in leaf phenolic content under low pH levels, an increase was actually obtained when combined with high temperature. This combination also increased fiber content but reduced leaf resistance, resulting in a net balance between leaf traits that increase susceptibility to grazing pressure and those that reduce it. Hence, we can conclude from this study that leaf trait responses can be highly variable, depending on whether driving environmental changes are considered as a single factor or in combination, and this will affect predictions of how these drivers will influence herbivory in ecosystems in the future.

In summary, the study shows how the main factors of globally and locally driven change in seagrass communities (warming, acidification and eutrophication) modify a broad spectrum of leaf traits, including the first set of empirical data on biomechanical traits. These changes led to an overall increase in plant palatability and, therefore, enhanced their consumption by a generalist herbivore *Paracentrotus lividus*, which could strengthen top-down effects in seagrass habitats and modify the fluxes of matter and energy in coastal areas. [78]. Predicting the net outcome of these co-occurring disturbances still remains a pressing challenge [79], and our findings suggest that more studies are needed to understand the direct and indirect impacts caused by change driven at the global and local scales and their effects on herbivory in seagrass communities.

## Supporting information

S1 Table Control measures for each treatment. Data are means ± SE. CpH: Current pH; FpH: Forecasted pH; ANH_4_^+^: Ambient NH_4_^+^; ENH_4_^+^: Enrichment NH_4_^+^.
(DOCX)

S2 Table Identification of phenolic compounds by UPLC-ESI-MS in *C*. *nodosa*. (DOCX)

S3 Table The statistics obtained from paired *t*-tests in consumption rate between *Ulva* sp. and *C*. *nodosa* in combined diet assays. Bold letters indicate significant differences.
(DOCX)

S1 Fig Chemical structures of compounds 1–7. (TIF)

S2 Fig Representative UPLC-MS chromatogram and selected MS/MS data of the detected compounds. Total ion current chromatogram obtained by UPLC-MS for a general extract obtained from *Cymodocea nodosa*. Numbers indicate the compounds detected. IS: internal standard used for quantification.
(TIF)

S3 Fig Main MS fragmentation of identified compounds. (TIF)
