# Supplementary material for: Global and local disturbances interact to modify seagrass palatability
Source: PLoS One. 2017 Aug 16;12(8):e0183256. doi: 10.1371/journal.pone.0183256 (PMC5558941; doi:10.1371/journal.pone.0183256)
Supplement: S1 Table — Data are means ± SE. CpH: Current pH; FpH: Forecasted pH; ANH4+: Ambient NH4+; ENH4+: Enrichment NH4+. (DOCX) [file pone.0183256.s001.docx]

| **Incubation treatments** | | | **NH_4_^+^  (μM) *** | **pH** | **pCO_2_ (ppm)** | **Temp. (ºC)** | **DIC (μmol kg** **^-1^)** |
| --- | --- | --- | --- | --- | --- | --- | --- |
| **Tª** | **pH** | **NH_4_^+^** |  |  |  |  |  |
| Local | CpH | ANH_4_^+^ | 0 | 8.14 ± 0.01 | 447 ± 21 | 21.93 ± 0.08 | 2358 ± 30 |
| Local | CpH | ENH_4_^+^ | 30.4 ± 1.3 | 8.13 ± 0.02 | 424 ± 22 | 21.94 ± 0.08 | 2179 ± 17 |
| Local | FpH | ANH_4_^+^ | 0 | 7.66 ± 0.01 | 744 ± 17 | 21.85 ± 0.05 | 1275 ± 22 |
| Local | FpH | ENH_4_^+^ | 31.8 ± 1.7 | 7.68 ± 0.01 | 750 ± 18 | 21.95 ± 0.08 | 1287 ± 30 |
| High | CpH | ANH_4_^+^ | 0 | 8.10 ± 0.01 | 412 ± 18 | 26.08 ± 0.02 | 2278 ± 23 |
| High | CpH | ENH_4_^+^ | 31.4 ± 1.5 | 8.10 ± 0.02 | 402 ± 19 | 26.07 ± 0.03 | 2214 ± 12 |
| High | FpH | ANH_4_^+^ | 0 | 7.66 ± 0.01 | 736 ± 12 | 26.10 ± 0.03 | 1210 ± 49 |
| High | FpH | ENH_4_^+^ | 32.1 ± 1.4 | 7.67 ± 0.01 | 729 ± 12 | 26.14 ± 0.03 | 1238 ± 15 |
