# Supplementary material for: Global and local disturbances interact to modify seagrass palatability
Source: PLoS One. 2017 Aug 16;12(8):e0183256. doi: 10.1371/journal.pone.0183256 (PMC5558941; doi:10.1371/journal.pone.0183256)
Supplement: S2 Table — (DOCX) [file pone.0183256.s002.docx]

| **Comp.** | ***t_R_ (min)*** | **Formula** | **MS^2^ focused on [M-H]^-^ (*m*/*z*)*** | **Identification** |
| --- | --- | --- | --- | --- |
| **1** | 2.57 | C_27_H_29_O_16_ | 609.1437 [M-H]^-^ (18), 301.0329 [M-H-308]^-^ (100) | ** |
| **2** | 2.67 | C_21_H_19_O_12_ | 463.0846 [M-H]^-^ (20), 301.0328 [M-H-162]^-^ (100) | ** |
| **3** | 2.80 | C_23_H_21_O_13_ | 505.0963 [M-H]^-^ (25), 463.0845 [M-H-42] ^-^ (10), 301.0325 [M-H-42-162]^-^ (100) | ref. 1 |
| **4** | 2.83 | C_28_H_31_O_16_ | 623.1577 [M-H]^-^ (35), 315.0502 [M-H-308]^-^ (100) | ref. 2 |
| **5** | 2.89 | C_22_H_21_O_12_ | 477.1010 [M-H]^-^ (15), 315.0510 [M-H-162]^-^ (100) | ref. 1 |
| **6** | 3.21 | C_26_H_29_O_10_ | 501.1773 [M-H]^-^ (38), 339.1230 [M-H-162]^-^ (100), 219.0645 [M-H-162-120]^-^ (30) | ref. 3, 4 |
| **7** | 3.84 | C_26_H_29_O_10_ | 663.2286 [M-H]^-^ (20), 501.1765 [M-H-162]^-^ (35), 339.1211 [M-H-162-162]^-^ (100), 219.0645 [M-H-162-162-120]^-^ (58) | ref. 3,5 |

*values in brackets represent relative abundance.

**standard available.

ref.1 a) Ma Y, Tanaka N, Vaniya A, Kind T, Fiehn O. Ultrafast Polyphenol Metabolomics of Red Wines Using MicroLC-MS/MS. Journal of Agricultural and food chemistry*.* 2016; 64: 505-512. b) Lin LZ, Sun J, Chen P, Harnly J. UHPLC-PDA-ESI/HRMS/MS^n^ Analysis of Anthocyanins, Flavonol Glycosides, and Hydroxycinnamic Acid Derivatives in Red Mustard Greens (*Brassica juncea* Coss Variety) Journal of Agricultural and food chemistry. 2011;59: 12059-12072.

Ref. 2 Mokrani A, Krisa S, Cluzet S, Da Costa G, Temsamani H, Renouf E, Mérillon JM, Madani K, Mesnil M, Monvoisin A. Phenolic contents and bioactive potential of peach fruit extracts. Food Chemistry. 2016; *202*, 212-220.

ref. 3 Quifer-Rada P, Vallverdú-Queralt A, Martínez-Huélamo M, Chiva-Blanch G, Jauregui O, Estruch R, Lamuela-Raventós R A comprehensive characterisation of beer polyphenols by high resolution mass spectrometry (LC-ESI-LTQ-Orbitrap-MS). Food Chemistry . 2015;169: 336-343.

ref. 4 Kim HY, Kim SH, Kang BY, Lee IS. Neuroprotective effects of medicinal plants *Arch. Pharm. Res.* **2008**; *31*: 1241-1246.

ref. 5 Arisawa M, Horiuchi T, Hayashi T, Tezuka Y, Kikuchi T, Morita N. Studies on constituents of *Evodia rutaecarpa* (Rutaceae). I. Constituents of the leaves. Chem. Pharm. Bull. 1993;41: 1472-1474.
