# Supplementary material for: Global and local disturbances interact to modify seagrass palatability
Source: PLoS One. 2017 Aug 16;12(8):e0183256. doi: 10.1371/journal.pone.0183256 (PMC5558941; doi:10.1371/journal.pone.0183256)
Supplement: S3 Table — Bold letters indicate significant differences. (DOCX) [file pone.0183256.s003.docx]

|  | **t** | **df** | ***p*-value** |
| --- | --- | --- | --- |
| Local Tª, CpH, ANH_4_^+^ | -7.568 | 4 | **0.0016** |
| Local Tª, CpH, ENH_4_^+^ | -3.978 | 4 | **0.016** |
| Local Tª, FpH, ANH_4_^+^ | -6.173 | 4 | **0.0034** |
| Local Tª, FpH, ENH_4_^+^ | -4.594 | 4 | **0.01** |
| High Tª, CpH, ANH_4_^+^ | -3.088 | 4 | **0.036** |
| High Tª, CpH, ENH_4_^+^ | -1.644 | 4 | **0.017** |
| High Tª, FpH, ANH_4_^+^ | -2.971 | 4 | **0.041** |
| High Tª, FpH, ENH_4_^+^ | -4.41 | 4 | **0.011** |
