# Supplementary figures and images for: Global and local disturbances interact to modify seagrass palatability
Source: PLoS One. 2017 Aug 16;12(8):e0183256. doi: 10.1371/journal.pone.0183256 (PMC5558941; doi:10.1371/journal.pone.0183256)

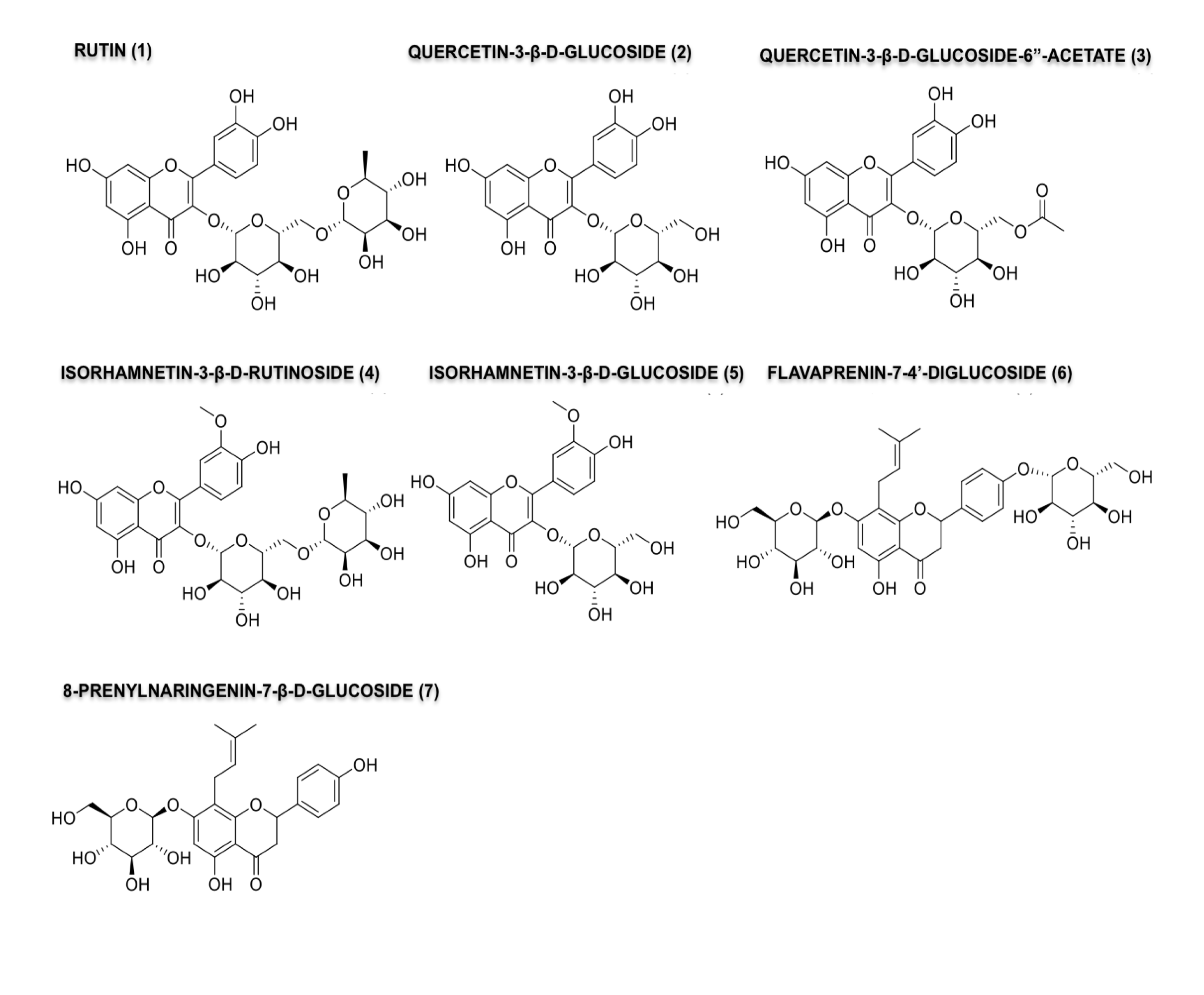

Supplement: S1 Fig — (TIF) [file pone.0183256.s004.tif]

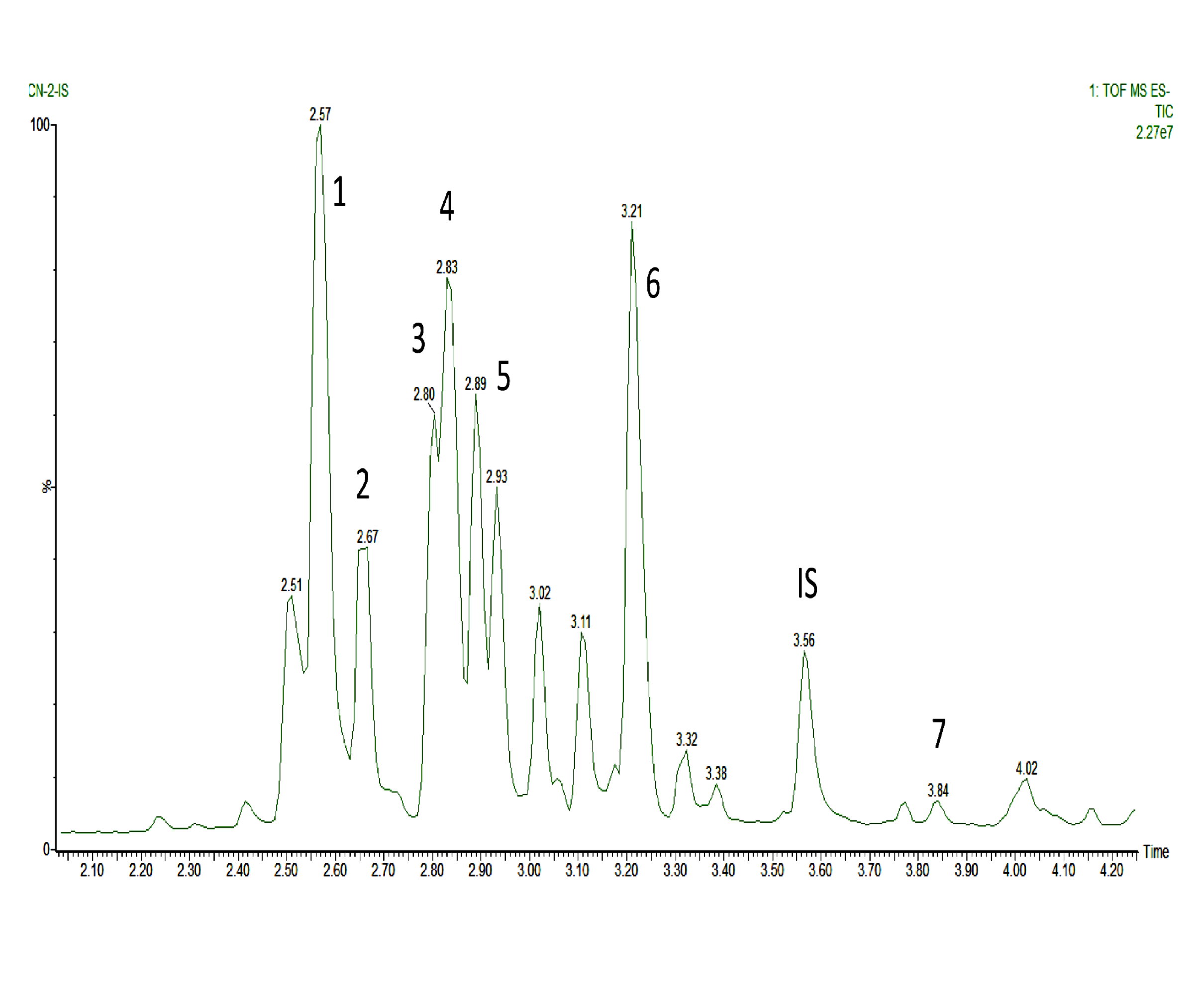

Supplement: S2 Fig — Total ion current chromatogram obtained by UPLC-MS for a general extract obtained from Cymodocea nodosa. Numbers indicate the compounds detected. IS: internal standard used for quantification. (TIF) [file pone.0183256.s005.tif]

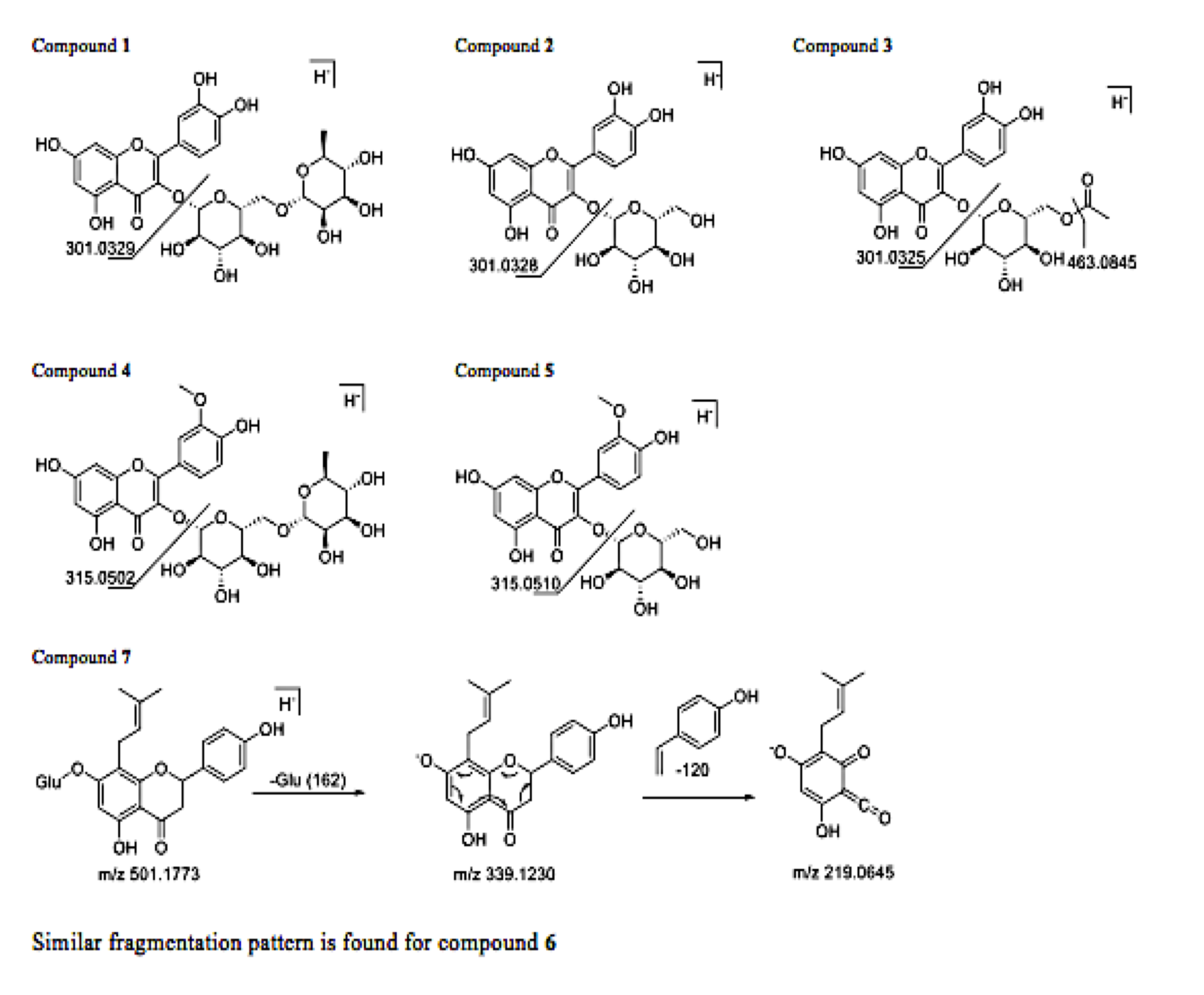

Supplement: S3 Fig — (TIF) [file pone.0183256.s006.tif]
